# Department-based need-supportive leadership and affective job satisfaction among Chinese university teachers: a cross-sectional serial mediation analysis of work-related basic psychological need satisfaction and work motivation

**DOI:** 10.3389/fpsyg.2026.1815184

**Published:** 2026-06-04

**Authors:** Xinyuan Li, Zhuoying Lin, Guanming Huang, Hanyang Cui

**Affiliations:** 1 School of Physical Education and Sports Science, Hengyang Normal University, Hengyang, Hunan, China; 2 School of Physical Education, Shihezi University, Shihezi, Xinjiang, China

**Keywords:** affective job satisfaction, department-based need-supportive leadership, self-determination theory, serial mediation, work motivation, work-related basic psychological need satisfaction

## Abstract

**Background:**

Guided by self-determination theory (SDT), we examined whether department-based need-supportive leadership, conceptualized as an SDT-based leadership behavior construct, is associated with affective job satisfaction among Chinese university teachers and whether work-related basic psychological need satisfaction and work motivation statistically account for this association through independent and serial indirect pathways.

**Methods:**

We surveyed in-service Chinese university teachers (*N* = 424). After controlling for gender, age, and academic rank, we tested a regression-based serial mediation model (PROCESS Model 6; 5,000 percentile bootstrap samples).

**Results:**

Department-based need-supportive leadership was positively associated with affective job satisfaction (*β*_total = 0.334, *p* < 0.001; total effect B = 0.4938, 95% CI [0.3625, 0.6252]). The direct effect remained significant after including work-related basic psychological need satisfaction and work motivation (*β* = 0.187, *p* < 0.001; c′ = 0.2758, 95% CI [0.1491, 0.4026]). The total indirect effect was 0.2180 (95% CI [0.1528, 0.2900]; 44.15%), with significant specific indirect effects via work-related basic psychological need satisfaction (B = 0.0583, 95% CI [0.0107, 0.1093]; 11.81%), via work motivation (*B* = 0.0856, 95% CI [0.0309, 0.1478]; 17.33%), and via the serial pathway through work-related basic psychological need satisfaction and work motivation (*B* = 0.0741, 95% CI [0.0492, 0.1053]; 15.01%).

**Conclusion:**

Department-based need-supportive leadership showed a positive association with teachers’ affective job satisfaction that was partially accounted for by indirect effect estimates via work-related basic psychological need satisfaction and work motivation. Given the cross-sectional, self-report design, findings should be interpreted as associations and indirect effect estimates rather than evidence of temporal ordering or causal mechanisms.

## 1 Introduction

According to the 2023 National Statistical Bulletin on the Development of Education released by the Ministry of Education of the People’s Republic of China, there were 3,074 higher education institutions nationwide, with 47,631,900 students enrolled in higher education and 2,074,900 full-time teachers (Ministry of Education of the People’s Republic of China, 2024). Against the backdrop of simultaneous growth in institutional scale and faculty size, academic departments serve as proximal units for allocating teaching and research tasks, coordinating resources, and managing day-to-day operations, and their governance and managerial interactions are therefore more directly reflected in university teachers’ work experiences. A report by UNESCO and the International Task Force on Teachers for Education 2030, using Australia as an illustrative case, notes that teachers who report higher satisfaction with work relationships also report a lower likelihood of leaving than those who report lower satisfaction, suggesting meaningful differences in retention risk by relationship-related satisfaction experiences (UNESCO and International Task Force on Teachers for Education 2030, 2024). Affective job satisfaction refers to individuals’ affective and experiential evaluations of their job, emphasizing feelings such as enjoyment, pleasure, and emotional identification, and is distinct from cognitively oriented job satisfaction that centers on rational appraisal (Thompson and Phua, 2012). In the present study, department-based need-supportive leadership is conceptualized as a self-determination theory-based leadership behavior construct rather than as a broad leadership style or a general perception of supervisor support; it refers to teachers’ overall perceptions of department leaders’ autonomy support, structure or competence support, and relatedness support in routine departmental governance (Deci et al., 2017; Tafvelin and Stenling, 2018). A meta-analysis in higher education also suggests a relatively stable, moderate positive correlation between leadership-related variables and academic staff job satisfaction (Kasalak et al., 2022). However, existing evidence has largely remained at the level of overall leadership-satisfaction correlations and has less often clarified how a department-level, need-supportive form of leadership, as specified by self-determination theory, is linked to affective job satisfaction through need satisfaction and motivational processes. More focused tests are still needed to clarify whether need-supportive leadership in departmental contexts can be statistically decomposed into distinct explanatory pathways and whether these pathways form a sequential pattern of indirect associations, particularly in samples of Chinese university teachers.

From the perspective of self-determination theory, need-supportive managerial contexts tend to co-occur with higher satisfaction of autonomy, competence, and relatedness needs at work, and need satisfaction is further associated with higher-quality motivational functioning and more positive experiences in attitudes and well-being (Deci et al., 2017). Thus, self-determination theory provides the primary rationale for linking department-based need-supportive leadership, work-related basic psychological need satisfaction, work motivation, and affective job satisfaction within one model. A meta-analysis in work settings provides robust correlational evidence consistent with the broader sequence of support, need satisfaction, motivation, and outcomes (Slemp et al., 2018). As a complementary work-design perspective, the motivational process articulated in the job demands-resources model emphasizes that job resources are linked to attitudinal outcomes through positive motivational processes such as motivation and engagement, offering an aligned process framework for understanding how supportive departmental contexts relate to job attitudes (Bakker et al., 2014). Building on these lines of theory, the present study sought to (1) examine whether department-based need-supportive leadership is positively associated with affective job satisfaction; (2) test whether work-related basic psychological need satisfaction and work motivation statistically account for this association through independent and serial indirect pathways; and (3) provide empirical evidence consistent with a structured conceptual framework linking need-supportive leadership, work-related basic psychological need satisfaction, work motivation, and affective job satisfaction, with implications for departmental governance in higher education. In doing so, the study extends prior leadership-satisfaction research by positioning department-based need-supportive leadership within a self-determination theory framework and by examining need satisfaction and work motivation as theoretically specified explanatory pathways. Because the study relies on cross-sectional survey data, the proposed paths are intended to characterize associations and estimates of indirect effects consistent with the theorized ordering, rather than to establish temporal precedence or causal mechanisms.

## 2 Literature review and research hypotheses

### 2.1 Department-based need-supportive leadership and affective job satisfaction

Department-based need-supportive leadership is treated in this study as a self-determination theory-based leadership behavior construct that captures university teachers’ perceptions of department leaders’ autonomy support, competence or structure support, and relatedness support in routine departmental governance (Deci et al., 2017; Tafvelin and Stenling, 2018), whereas affective job satisfaction reflects teachers’ affective satisfaction with their work. From an SDT perspective, such leadership represents a proximal need-supportive social context. When department leaders acknowledge teachers’ perspectives, provide clear structure and competence-related feedback, coordinate resources, and maintain respectful interpersonal communication, these behaviors are theoretically expected to be associated with more favorable affective evaluations of work. Empirical findings are broadly consistent with this expectation. Meta-analytic evidence in higher education suggests a relatively stable, moderate positive correlation between leadership-related variables and academic staff job satisfaction (Kasalak et al., 2022). Large-scale, multi-country working-conditions surveys similarly show that employees’ perceived supportive supervisory behaviors are positively associated with job satisfaction (García-Cabrera et al., 2023). At the departmental governance level, more favorable evaluations of department chairs have been observed alongside higher job satisfaction among faculty (Bäker and Goodall, 2020), and studies across different university samples have reported positive links between supportive leadership styles and faculty job satisfaction (Alonderienė and Majauskaitė, 2016). In addition, a meta-analysis of empowering leadership indicates overall positive associations between empowering leadership and subordinates’ work attitudes (Kim et al., 2018). Notably, although some of these studies use global job satisfaction measures, the affective satisfaction components embedded in such measures overlap conceptually with affective job satisfaction in the present study and thus provide directional support for our expectations. Taken together, SDT-based reasoning and prior empirical evidence support the following hypothesis:

Hypothesis 1: Department-based need-supportive leadership is positively associated with affective job satisfaction.

### 2.2 Mediating role of work-related basic psychological need satisfaction

One proposed pathway in the present study concerns the mediating role of work-related basic psychological need satisfaction, which may account, at a statistical level, for part of the association between department-based need-supportive leadership and affective job satisfaction. Work-related basic psychological need satisfaction reflects the extent to which teachers experience autonomy, competence, and relatedness need satisfaction at work. According to basic psychological needs theory, social contexts that support autonomy, competence, and relatedness are expected to be associated with higher satisfaction of these needs (Deci et al., 2017). Because department-based need-supportive leadership is organized around autonomy support, competence or structure support, and relatedness support, it maps directly onto teachers’ work-related basic psychological need satisfaction. Prior research suggests that supportive or servant leadership tends to be positively associated with need satisfaction, and that need satisfaction can account for leadership-related differences in employees’ positive states within statistical models (Jiang and Wei, 2024). At the job design level, process models and review evidence have also supported positive links between job resources and need satisfaction, as well as corresponding associations between need satisfaction and more favorable work attitudes (Trépanier et al., 2015; Manganelli et al., 2018). Regarding the need satisfaction-job satisfaction link, evidence from healthcare samples indicates that autonomy or relatedness need satisfaction is positively associated with job satisfaction (Hood and Patton, 2022), and studies of Chinese physicians similarly report positive links between work-related basic psychological need satisfaction and job satisfaction within models that include indirect association patterns (Xu et al., 2022). Complementary evidence from needs–supplies fit research also suggests that closer alignment between employees’ needs and workplace supplies is associated with higher job satisfaction (Dörendahl et al., 2020). Consistent with this line of evidence, a three-wave study in scientific teams modeled needs–supplies fit as a mediator linking transformational leadership to job satisfaction, while also considering work-related strain as an additional indicator of well-being (Klaic et al., 2018). Taken together, SDT-based reasoning and prior empirical evidence suggest that higher work-related basic psychological need satisfaction is expected to be positively associated with affective job satisfaction and may provide a theoretically meaningful indirect pathway linking department-based need-supportive leadership with affective job satisfaction.

Hypothesis 2: Work-related basic psychological need satisfaction mediates the positive association between department-based need-supportive leadership and affective job satisfaction.

### 2.3 Mediating role of work motivation

A second proposed pathway concerns the mediating role of work motivation, which may statistically account for part of the association between department-based need-supportive leadership and affective job satisfaction. Work motivation refers to individuals’ motivational functioning at work, including their willingness to invest effort and sustain engagement, as well as qualitative differences in motivation. From an SDT perspective, need-supportive work contexts are expected to be associated with more favorable motivational functioning because support for autonomy, competence, and relatedness is theoretically linked to volitional engagement, perceived effectiveness, and internalization of work-related goals (Deci et al., 2017). Accordingly, department-based need-supportive leadership, as reflected in supportive involvement in decision-making, competence-related guidance, resource coordination, and respectful communication, is expected to be positively associated with work motivation among university teachers. As a supplementary leadership-process perspective, path–goal theory also suggests that supportive leadership behaviors, such as goal clarification, barrier removal, and resource coordination, are consistent with stronger motivational functioning. Empirical findings provide broadly consistent support for this expectation. For example, in a cross-sectional study of Belgian nurses, engaging leadership was associated with higher work engagement and lower burnout, and these associations were consistent with indirect associations via perceived job resources and intrinsic motivation in a serial modeling framework (Kohnen et al., 2024). Field intervention research also reports that after supervisors receive need-support training, employees report higher autonomous motivation and lower amotivation (Kaabomeir et al., 2023), offering stronger empirical grounding for the expectation that leadership support and motivation indicators vary in the same direction. It should be noted that some studies operationalize higher-quality motivation using autonomous or intrinsic motivation, whereas work motivation in the present study can be understood as a broader indicator of motivational functioning. Thus, the available evidence provides directional support for our work motivation hypothesis. As a complementary work-design perspective, the motivational process of the job demands–resources model also treats motivation as a key link between job resources and positive job attitudes. A meta-analysis in the teaching domain shows that autonomous teacher motivation is positively associated with job satisfaction, which includes affective satisfaction components, and path models are consistent with a sequence in which need satisfaction is linked to satisfaction and well-being through autonomous motivation (Slemp et al., 2020). In addition, perceived mission command, conceptualized as an autonomy-supportive leadership approach, showed an indirect association with autonomous motivation via autonomy need satisfaction, and autonomous motivation was positively associated with job satisfaction (Knevelsrud et al., 2024), providing further alignment with a leadership–motivation–satisfaction pathway. Taken together, SDT-based reasoning and prior empirical evidence suggest that work motivation may provide a theoretically meaningful indirect pathway linking department-based need-supportive leadership with affective job satisfaction.

Hypothesis 3: Work motivation mediates the positive association between department-based need-supportive leadership and affective job satisfaction.

### 2.4 Serial mediation effect of work-related basic psychological need satisfaction and work motivation

The proposed serial pathway is grounded primarily in self-determination theory. In this framework, department-based need-supportive leadership represents a proximal need-supportive social context, work-related basic psychological need satisfaction reflects teachers’ satisfaction of autonomy, competence, and relatedness needs at work, and work motivation reflects subsequent motivational functioning. Within self-determination theory, basic psychological needs theory supports the first segment of the proposed sequence by linking need-supportive contexts with satisfaction of autonomy, competence, and relatedness needs, whereas organismic integration theory supports the next segment by explaining how need satisfaction is linked to motivational internalization and higher-quality motivational functioning (Deci et al., 2017; Van den Broeck et al., 2016). Meta-analytic, dyadic, and longitudinal studies across contexts have documented aligned patterns among leadership support, need satisfaction, and autonomous motivation (Slemp et al., 2018; Chiniara and Bentein, 2016; Olafsen et al., 2018). Related work-characteristics and leadership process models have similarly placed need satisfaction as a proximal explanatory link between contextual resources and subsequent outcomes (Trépanier et al., 2015; Sedlářík et al., 2024). These findings provide theoretical and empirical grounding for the proposed serial order, although much of the existing evidence relies on correlational designs and is therefore best interpreted as directional support rather than direct evidence for temporal precedence or causal mechanisms.

At the later stage of the sequence, meta-analytic work on motivation types indicates that autonomous motivation shows the strongest links with attitudinal indicators such as satisfaction, whereas amotivation is strongly associated with unfavorable outcomes (Van den Broeck et al., 2021). Two-wave evidence further suggests that need satisfaction can be linked to satisfaction and well-being through domain-specific autonomous motivation and related authenticity processes, particularly in the work domain (White et al., 2024). In addition, evidence from Chinese occupational samples has also observed positive path structures connecting work-related basic psychological need satisfaction, work motivation, and job satisfaction (Xu et al., 2022). Together, SDT-based reasoning and prior empirical evidence support examining whether work-related basic psychological need satisfaction and work motivation jointly account for part of the association between department-based need-supportive leadership and affective job satisfaction through a sequential indirect pathway.

Hypothesis 4: Work-related basic psychological need satisfaction and work motivation serially mediate the positive association between department-based need-supportive leadership and affective job satisfaction.

**Figure 1:**
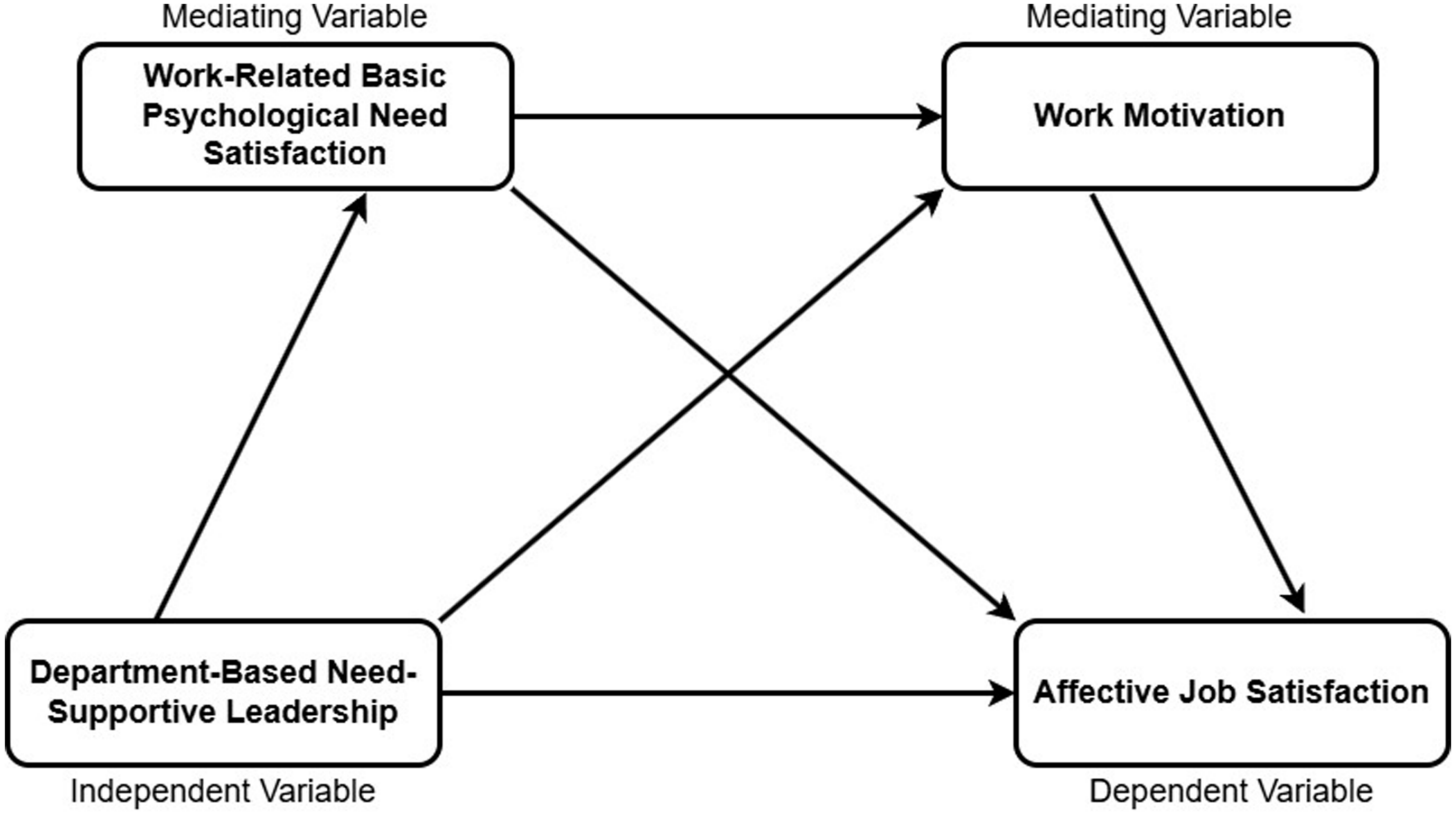
Conceptual framework of the proposed serial mediation model. Department-based need-supportive leadership is modeled as the independent variable, work-related basic psychological need satisfaction and work motivation as mediators, and affective job satisfaction as the dependent variable.

The proposed serial order is theory driven, and alternative orderings or competing models are not systematically compared. In sum, prior research suggests a stable positive association between department-based need-supportive leadership and affective job satisfaction and indicates that work-related basic psychological need satisfaction and work motivation may contribute independent and sequential indirect pathways. Building on this theoretical and empirical foundation, we developed a conceptual framework (Figure 1) to (1) examine the positive association between department-based need-supportive leadership and affective job satisfaction, (2) test the mediating role of work-related basic psychological need satisfaction in this association, (3) test the mediating role of work motivation in this association, and (4) test the serial mediation of work-related basic psychological need satisfaction and work motivation in the association between department-based need-supportive leadership and affective job satisfaction.

## 3 Methods

### 3.1 Participants

This study used a cross-sectional survey design and recruited participants through non-probability convenience sampling among in-service university teachers at Hengyang Normal University, Hunan, China. The study was designed as an initial theory-driven test of the proposed self-determination theory-based association structure rather than as a nationally representative survey of Chinese university teachers. The single-institution setting provided a relatively consistent institutional and governance context in which to examine the focal associations among department-based need-supportive leadership, work-related basic psychological need satisfaction, work motivation, and affective job satisfaction. Accordingly, the sample should not be interpreted as representative of all Chinese university teachers; differences in institutional type, regional context, resource structure, and departmental governance may affect the observed associations, as discussed further in the Limitations and future directions section. An online questionnaire was distributed via university- and department-level work-group channels from January 16 to January 25, 2026, with 500 questionnaires disseminated in total. Before responding, participants read an online informed consent statement indicating that participation was entirely voluntary and that they could withdraw at any time; the survey was completed anonymously, no personally identifiable information was collected, and data were used solely for academic research and reported in aggregated form. After data collection, responses were screened and cleaned using prespecified data-quality rules. Exclusion criteria were as follows: (1) not an in-service university teacher, (2) incomplete questionnaire or missing key items, (3) unusually short completion time, (4) evident response patterning (e.g., long strings of identical responses across consecutive items) indicating poor response differentiation, and (5) inconsistent or logically conflicting answers. The final analytic sample comprised 424 participants (valid response rate: 84.80%). The sample included 225 men (53.07%) and 199 women (46.93%). Age was recorded in four groups: 30 years or younger (n = 110), 31 to 40 years (n = 120), 41 to 50 years (n = 105), and 51 years or older (n = 89). Academic rank was collapsed into four levels (junior, intermediate, associate senior, and senior), with 95, 135, 120, and 74 participants in each category, respectively; this consolidation covered teaching, research, and laboratory or technical tracks and facilitated subsequent group comparisons and covariate adjustment. The study protocol was approved by the Ethics Committee of Hengyang Normal University (IRB No.: 202611002).

### 3.2 Measurement

#### 3.2.1 Need support at work scale

Department-based need-supportive leadership was measured using the semantically equivalent Chinese translation of the Need Support at Work Scale (Tafvelin and Stenling, 2018). Grounded in self-determination theory, this instrument assesses university teachers’ perceptions of their department leaders’ need-supportive behaviors across three dimensions: autonomy support, competence (structure) support, and relatedness support. The scale contains 12 items (three subscales with four items each) rated on a 5-point frequency-based Likert scale (1 = never or almost never, 5 = always), with higher scores indicating higher perceived need support. An example item is “Tries to understand my perspective before stating his/her opinion.” (autonomy support). In the present sample (*N* = 424), Cronbach’s alpha values were 0.817 for autonomy support, 0.858 for competence (structure) support, and 0.819 for relatedness support. Confirmatory factor analysis indicated acceptable model fit (χ^2^/df = 2.929, RMSEA = 0.068, CFI = 0.955, TLI = 0.941), supporting the scale’s factorial validity and reliability for subsequent analyses.

#### 3.2.2 Work-related basic psychological need satisfaction

Work-related basic psychological need satisfaction was assessed using the semantically equivalent Chinese translation of the Work-Related Basic Psychological Need Satisfaction scale (Van den Broeck et al., 2010), which includes three dimensions: autonomy need satisfaction, competence need satisfaction, and relatedness need satisfaction. Items were rated on a 5-point Likert scale (1 = strongly disagree, 5 = strongly agree), with higher scores indicating higher need satisfaction; some items were reverse scored. An example item is “I do not feel connected with other people.” (reverse scored, relatedness need). In the present sample (*N* = 424), Cronbach’s alpha values were 0.904 for autonomy, 0.891 for competence, and 0.894 for relatedness. Confirmatory factor analysis showed excellent fit (χ^2^/df = 1.020, RMSEA = 0.007, CFI = 0.999, TLI = 0.999), supporting the scale’s validity and reliability in this sample.

#### 3.2.3 Multidimensional work motivation scale

Work motivation was measured using the semantically equivalent Chinese translation of the Multidimensional Work Motivation Scale (Gagné et al., 2015). The instrument covers six types of motivational regulation: amotivation, external regulation (material), external regulation (social), introjected regulation, identified regulation, and intrinsic motivation, comprising 19 items in total. To align with the scoring format of the present survey and reduce respondent burden, the original 7-point response format was adapted to a 5-point Likert scale (1 = strongly disagree, 5 = strongly agree); therefore, scores from this study are not directly comparable in magnitude to findings from studies using the original 7-point format. An example item is “I don’t, because I really feel that I’m wasting my time at work.” (amotivation). In the present sample (*N* = 424), Cronbach’s alpha values for the six dimensions were 0.779, 0.814, 0.778, 0.832, 0.778, and 0.774, respectively. Confirmatory factor analysis indicated good fit (χ^2^/df = 1.151, RMSEA = 0.019, CFI = 0.992, TLI = 0.991), suggesting that the expected measurement structure and internal consistency were retained under the 5-point response format.

#### 3.2.4 Brief index of affective job satisfaction

Affective job satisfaction was measured using the semantically equivalent Chinese translation of the Brief Index of Affective Job Satisfaction (Thompson and Phua, 2012). This single-factor scale contains four items rated on a 5-point Likert scale (1 = strongly disagree, 5 = strongly agree). The affective job satisfaction score was computed as the mean of the four items, with higher scores indicating higher affective job satisfaction. An example item is “I find real enjoyment in my job.” In the present sample (*N* = 424), Cronbach’s alpha was 0.806, standardized factor loadings ranged from 0.671 to 0.802, CR was 0.808, and the AVE was 0.514, supporting acceptable internal consistency and convergent validity for subsequent analyses.

### 3.3 Statistical analysis

Data were organized and analyzed using SPSS 26.0, and measurement models were evaluated using AMOS 26. After reverse scoring items as required by each instrument, composite scores were computed as item means. Means and standard deviations were calculated for department-based need-supportive leadership, work-related basic psychological need satisfaction, work motivation, and affective job satisfaction, and Pearson correlations were used to describe associations among variables. For demographic group comparisons, independent-samples t tests were used for gender, and one-way analyses of variance were used for age group and academic rank; when homogeneity of variance was violated, the gender difference in affective job satisfaction was examined using Welch’s t test. Common method variance was diagnostically examined using Harman’s single-factor test by entering all items from the four constructs into an unrotated principal component analysis and using the variance explained by the first factor as a diagnostic indicator. Hypotheses were examined within a regression and mediation modeling framework. Gender, age group, and academic rank were included as covariates in all regression equations to account for potential demographic differences in parameter estimates. Standardized coefficients (*β*) and corresponding t values were reported for regression paths, along with the explained variance (*R^2^*) for each equation. Serial mediation was tested using the PROCESS macro (Model 6). Because the data were cross-sectional, the mediation analyses were used to estimate theory-consistent statistical indirect effects rather than to establish causal mediation or temporal ordering. After controlling for covariates, the total effect (c), direct effect (c′), and specific indirect effects were estimated, and percentile bootstrap confidence intervals were obtained using 5,000 resamples; an indirect effect estimate was considered statistically distinguishable from zero when the 95% confidence interval did not include zero. The proportion of the total effect accounted for by each indirect effect was also reported to describe relative contributions. To maintain consistent reporting, standardized coefficients were used for path estimates, whereas effect decomposition and indirect effects were reported as unstandardized effects (B) with bootstrap 95% confidence intervals. At the measurement level, confirmatory factor analysis was first conducted in AMOS 26 to provide evidence for the measurement structure and construct validity of the study variables; composite scores were then used as observed variables in the regression and PROCESS analyses. This two-step approach allowed measurement validation to be established while using the PROCESS framework to obtain directly comparable estimates of total, direct, and specific indirect effects and their relative contributions within a feasible and interpretable analytic strategy. All tests were two-tailed, with statistical significance set at *p* < 0.05.

## 4 Results

### 4.1 Descriptive statistics for department-based need-supportive leadership, work-related basic psychological need satisfaction, work motivation, and affective job satisfaction

Table 1 presents descriptive statistics for department-based need-supportive leadership (DBNSL), work-related basic psychological need satisfaction (W-BNS), work motivation (WM), and affective job satisfaction (AJS). The overall mean (SD) scores were 2.98 (0.52), 3.01 (0.58), 3.00 (0.50), and 2.99 (0.77), respectively. Gender comparisons showed significant differences for all four variables (|*t*| = 2.507–2.665, *p*s < 0.05): men reported higher scores for department-based need-supportive leadership and work motivation, whereas women reported higher scores for work-related basic psychological need satisfaction and affective job satisfaction. One-way analyses of variance further indicated significant differences by age group and academic rank across all four variables (*F*s = 3.674–4.128, *p*s < 0.05). These group differences provided a rationale for controlling gender, age, and academic rank in subsequent regression and mediation analyses.

**Table 1:** Descriptive statistics (M ± SD) and group difference tests for DBNSL, W-BNS, WM, and AJS.

Group	***N***	Department-Based Need-Supportive Leadership (DBNSL)	Work-Related Basic Psychological Need Satisfaction (W-BNS)	Work Motivation (WM)	Affective Job Satisfaction (AJS)
Male	225	3.04 ± 0.53	2.94 ± 0.59	3.06 ± 0.49	2.89 ± 0.71
Female	199	2.92 ± 0.50	3.09 ± 0.57	2.94 ± 0.50	3.09 ± 0.83
Overall	424	2.98 ± 0.52	3.01 ± 0.58	3.00 ± 0.50	2.99 ± 0.77
Gender differences (*t*)		2.507*	2.665**	2.621**	2.664**
Age differences (*F*)		3.737*	3.970**	3.777*	3.674*
Academic rank differences (*F*)		3.728*	3.898**	4.128**	3.820*

Values are presented as M ± SD. Gender differences were examined using independent-samples *t*-tests, and age and academic rank differences were examined using one-way ANOVAs. *t* values are reported as absolute values. For AJS, Welch’s *t*-test was used due to unequal variances. *p < 0.05, **p < 0.01, ***p < 0.001.

### 4.2 Assessment of common method bias

Common method variance (CMV) was diagnostically examined using Harman’s single-factor test. All items from the four constructs were entered simultaneously into an unrotated principal component analysis (PCA). Thirteen factors with eigenvalues greater than 1 were extracted, accounting for 68.374% of the total variance. The first factor accounted for 21.643% of the variance, which is below the commonly used heuristic benchmark of 40% often reported when applying Harman’s test (Chen, 2025), suggesting that the covariance structure was unlikely to be dominated by a single common method factor. Nevertheless, Harman’s single-factor test is a coarse, *post hoc* diagnostic and is not a definitive test of CMV; methodological work has emphasized that CMV may still be present even when the first-factor share is below such heuristic cutoffs (Podsakoff et al., 2003; Baumgartner et al., 2021). Accordingly, results should be interpreted in light of the cross-sectional, self-report design.

### 4.3 Correlation analysis of department-based need-supportive leadership, work-related basic psychological need satisfaction, work motivation, and affective job satisfaction

Table 2 reports the means, standard deviations, and Pearson correlation coefficients for department-based need-supportive leadership, work-related basic psychological need satisfaction, work motivation, and affective job satisfaction (two-tailed tests, *N* = 424). Department-based need-supportive leadership was positively correlated with work-related basic psychological need satisfaction (*r* = 0.339, *p* < 0.001), work motivation (*r* = 0.285, p < 0.001), and affective job satisfaction (*r* = 0.310, *p* < 0.001). Work-related basic psychological need satisfaction was positively correlated with work motivation (*r* = 0.373, *p* < 0.001) and affective job satisfaction (*r* = 0.359, *p* < 0.001). Work motivation was also positively correlated with affective job satisfaction (*r* = 0.446, *p* < 0.001). These correlations provided correlational support for the subsequent regression models and indirect effect tests.

**Table 2:** Means, standard deviations, and correlations among study variables.

**Variable**	**M**	**SD**	**1**	**2**	**3**	**4**
Department-based need-supportive leadership (DBNSL)	2.98	0.52	1			
Work-related basic psychological need satisfaction (W-BNS)	3.01	0.58	0.339***	1		
Work motivation (WM)	3.00	0.50	0.285***	0.373***	1	
Affective job satisfaction (AJS)	2.99	0.77	0.310***	0.359***	0.446***	1

*N* = 424. Pearson correlations (two-tailed) are reported. ***p < 0.001.

### 4.4 Testing the mediating effects of work-related basic psychological need satisfaction and work motivation in the association between department-based need-supportive leadership and affective job satisfaction

A serial mediation model was tested with department-based need-supportive leadership as the predictor, work-related basic psychological need satisfaction and work motivation as mediators, and affective job satisfaction as the outcome. After controlling for gender, age, and academic rank, PROCESS Model 6 was used to test the serial mediation model, and 95% confidence intervals for the indirect effects were estimated using 5,000 percentile bootstrap resamples (*N* = 424). In parallel, a confirmatory factor analysis of the four-construct measurement model was conducted in AMOS 26 and showed good fit (χ^2^/df = 1.178, RMSEA = 0.021, CFI = 0.978, TLI = 0.976). Regression results are presented in Table 3 (standardized coefficients, *β*). Department-based need-supportive leadership was positively associated with work-related basic psychological need satisfaction (*β* = 0.359, *t* = 7.916, *p* < 0.001, *R*^2^ = 0.154). When work motivation was the outcome, both department-based need-supportive leadership (*β* = 0.145, *t* = 3.084, *p* < 0.01) and work-related basic psychological need satisfaction (*β* = 0.351, *t* = 7.408, *p* < 0.001) showed positive associations with work motivation (*R*^2^ = 0.206). When affective job satisfaction was the outcome, work motivation (*β* = 0.398, *t* = 8.896, *p* < 0.001), work-related basic psychological need satisfaction (*β* = 0.110, *t* = 2.385, *p* < 0.05), and department-based need-supportive leadership (*β* = 0.187, *t* = 4.278, *p* < 0.001) were each positively associated with affective job satisfaction (*R*^2^ = 0.336). Without the mediators included, the total effect of department-based need-supportive leadership on affective job satisfaction was also significant (*β*_total = 0.334, *t* = 7.389, *p* < 0.001), consistent with H1.

**Table 3:** Regression analysis of the serial mediation model.

**Variable**	**W-BNS**		**WM**		**AJS**		**Overall effect**	
	*β*	*t*	*β*	*t*	*β*	*t*	*β*	*t*
DBNSL	0.359	7.916***	0.145	3.084**	0.187	4.278***	0.334	7.389***
W-BNS	—	—	0.351	7.408***	0.110	2.385*	—	—
WM	—	—	—	—	0.398	8.896***	—	—
*R* ^2^	0.154		0.206		0.336		0.157	
*F*	19.115***		21.673***		35.123***		19.474***	

Standardized coefficients (β) are reported. Gender, age, and academic rank were included as covariates in all models. *p < 0.05, **p < 0.01, ***p < 0.001. Unstandardized effects (B) for the total, direct, and indirect effects are reported in Table 4.

**Figure 2:**
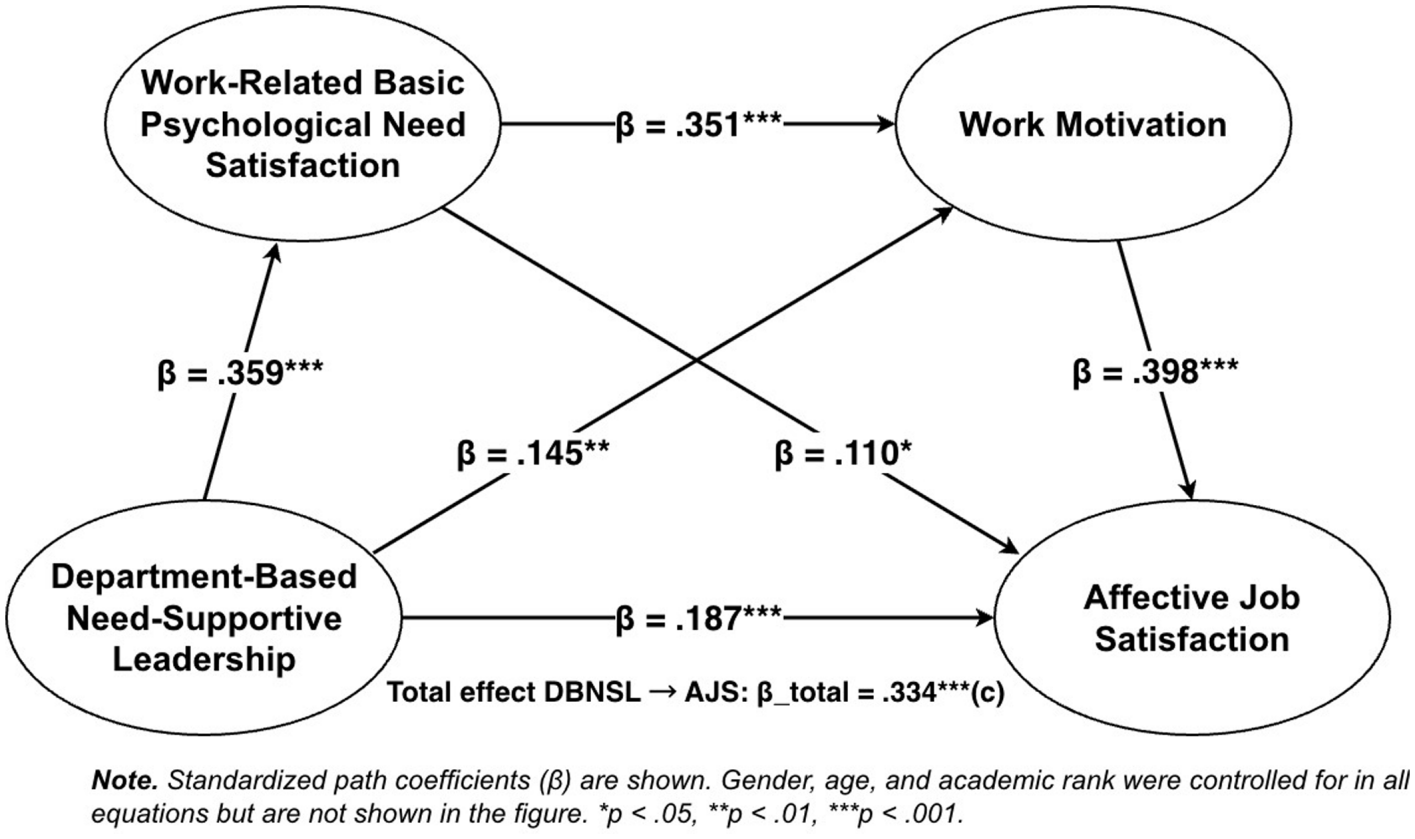
Standardized path coefficients of the serial mediation model. Gender, age, and academic rank were controlled for in all equations but are not shown in the figure. The total effect (c) was estimated in a separate model without mediators. *p < 0.05, **p < 0.01, ***p < 0.001.

Indirect effect estimates are summarized in Table 4 (unstandardized effects, B, with bootstrap 95% confidence intervals). The total effect estimate of department-based need-supportive leadership on affective job satisfaction was *B* = 0.4938 (95% CI [0.3625, 0.6252]). After including work-related basic psychological need satisfaction and work motivation, the direct effect estimate remained significant (c′ = 0.2758, 95% CI [0.1491, 0.4026]). The total indirect effect was 0.2180 (95% CI [0.1528, 0.2900]), accounting for 44.15% of the total effect. All three specific indirect effects were significant: DBNSL → W-BNS → AJS (*B* = 0.0583, 95% CI [0.0107, 0.1093], 11.81%), DBNSL → WM → AJS (*B* = 0.0856, 95% CI [0.0309, 0.1478], 17.33%), and DBNSL → W-BNS → WM → AJS (B = 0.0741, 95% CI [0.0492, 0.1053], 15.01%). These results were consistent with H2, H3, and H4, and the standardized path coefficients are illustrated in Figure 2. Given the cross-sectional survey design, mediation and serial mediation analyses were used to estimate statistical indirect effects; findings should be interpreted as associations and indirect effect estimates rather than evidence of temporal ordering or causal mechanisms.

**Table 4:** Mediating effect test and effect size.

**Path**	**Unstandardized effect (B)**	**Proportion of total effect**	**95% CI**	
			**LL**	**UL**
DBNSL → W-BNS → AJS	0.0583	11.81%	0.0107	0.1093
DBNSL → WM → AJS	0.0856	17.33%	0.0309	0.1478
DBNSL → W-BNS → WM → AJS	0.0741	15.01%	0.0492	0.1053
Total indirect	**0.2180**	**44.15%**	**0.1528**	**0.2900**
Direct effect (c′)	0.2758	—	0.1491	0.4026
Total effect (c)	0.4938	—	0.3625	0.6252

Unstandardized effects (B) are reported. Indirect effects were tested using PROCESS Model 6 with 5,000 percentile bootstrap samples and 95% confidence intervals. Gender, age, and academic rank were included as covariates. Proportions are calculated relative to the total effect (c) and are presented as percentages. LL and UL are reported to four decimal places. *N* = 424. Bold values indicate the total indirect effect row, including the total indirect effect, its proportion of the total effect, and the corresponding 95% confidence interval.

## 5 Discussion

This study examined the association structure among department-based need-supportive leadership (DBNSL), work-related basic psychological need satisfaction (W-BNS), work motivation (WM), and affective job satisfaction (AJS) in a sample of Chinese university teachers, with AJS capturing the affective facet of satisfaction with work experiences. Overall, the findings supported a partial mediation pattern: DBNSL was positively associated with AJS, and W-BNS and WM provided statistically significant independent and serial indirect pathways. The direct association between DBNSL and AJS remained significant after both mediators were included, indicating that need satisfaction and work motivation accounted for part, but not all, of this association. Because the indirect portion was distributed across the three pathways, no single pathway should be interpreted as a dominant explanation. Given the cross-sectional, self-report survey design, the findings are interpreted as theory-consistent associations and statistical indirect effect estimates rather than evidence of temporal precedence or causal mechanisms.

### 5.1 Departmental need support as a proximal context for affective job satisfaction

After controlling for gender, age, and academic rank, department-based need-supportive leadership was positively associated with affective job satisfaction. When work-related basic psychological need satisfaction and work motivation were included, this association retained a significant direct component, indicating that W-BNS and WM provided only a partial statistical account of the DBNSL-AJS association. From an SDT perspective, this result suggests that department-level support for teachers’ autonomy, competence or structure, and relatedness can be understood as a proximal social-contextual condition associated with more favorable affective evaluations of work. This pattern aligns with prior evidence linking need-supportive or autonomy-supportive leadership with job satisfaction. Meta-analytic evidence, for example, indicates a robust positive association between leader autonomy support and job satisfaction (Slemp et al., 2018). In educational systems, perceived autonomy support from administrators has been reported to be positively associated with job satisfaction (Chang et al., 2015), and teacher-focused research has examined leadership styles alongside job satisfaction and related outcomes within similar correlational frameworks (Haerens et al., 2022). In higher education and knowledge-work contexts, positive links between empowering leadership and academic staff job satisfaction have been reported and discussed in relation to leader-member exchange and trust (Horoub and Zargar, 2022), and inclusive leadership has been associated with job satisfaction among young university employees, with work-family balance examined as a differentiating pathway (Li and Zhou, 2023). Evidence from public-sector and service-industry samples similarly documents positive associations between supportive or servant leadership and job satisfaction (Coun et al., 2023; Uman et al., 2024). Consistent with this broader pattern, a large preregistered study further found that manager autonomy support was positively associated with job satisfaction within a job demands-resources framework (DeHaan et al., 2024). In a Chinese service sector context, the co-occurrence among perceived supervisor autonomy support, autonomous motivation, and job satisfaction has also been supported (Zhang et al., 2023). The remaining direct component suggests that the DBNSL-AJS association may also involve work-context factors beyond W-BNS and WM, such as leader-member exchange quality, organizational justice, resource availability, job autonomy, and psychological safety. From a departmental governance standpoint, this finding indicates that need-supportive behaviors are relevant to teachers’ affective work experiences because they are enacted through routine practices such as task allocation, feedback, communication, and resource coordination. Given the cross-sectional, self-report design, these interpretations are limited to theory-consistent associations rather than evidence of causal mechanisms or temporal ordering.

### 5.2 Basic psychological need satisfaction as a proximal explanatory pathway

Within the serial mediation model, W-BNS showed an independent and statistically significant indirect pathway in the association between DBNSL and AJS. The corresponding path pattern indicated that DBNSL was positively associated with W-BNS and that W-BNS remained positively associated with AJS after WM was included, suggesting that need satisfaction provided a partial statistical account of the DBNSL-AJS association. This pattern is consistent with prior findings on both segments of the proposed linkage, namely, need-supportive or autonomy-supportive leadership in relation to need satisfaction, and need satisfaction in relation to job satisfaction or affective job satisfaction. For example, two-wave research has documented positive links between need-supportive leadership behaviors and basic psychological need satisfaction, with need satisfaction used to account for differences in important work outcomes within statistical models (Stenling et al., 2024). Public sector research has similarly positioned leadership’s motivational relevance at the point where autonomy, competence, and relatedness needs are satisfied (Jensen and Bro, 2018). Cross-national and cross-level studies also report that engaging leadership, servant leadership, and authentic leadership are linked with positive employee states and performance through basic psychological need satisfaction as a key intermediary (Rahmadani et al., 2019; Chiniara and Bentein, 2016; Leroy et al., 2015). On the need satisfaction-to-satisfaction segment, organizational research has explicitly described basic psychological need satisfaction as a proximal source of job satisfaction (Van den Broeck et al., 2016) and a core condition for favorable work attitudes in the self-determination theory tradition (Deci et al., 2017). Public sector experimental work has also linked manipulated differences in need satisfaction with differences in affective job satisfaction (Battaglio et al., 2022). Two-wave survey evidence and studies in Chinese university teacher samples likewise show a stable positive association between need satisfaction at work and job satisfaction (Busque-Carrier et al., 2022; Xiong et al., 2015). In basic psychological needs theory terms, DBNSL reflects proximal need-supportive inputs at the departmental level, whereas W-BNS reflects teachers’ experienced satisfaction of autonomy, competence, and relatedness needs at work. Such inputs include providing actionable choice and participation opportunities in decisions and task assignments, offering clear and respectful competence-related feedback and coordinating resources for teaching and research, and maintaining care and acceptance in everyday interactions. These features are expected to align with higher autonomy, competence, and relatedness need satisfaction at work, as reflected in higher W-BNS. As a complementary interpretation, W-BNS can also be understood as capturing the perceived match between environmental supplies and individuals’ psychological needs. A closer match tends to coincide with a profile characterized by more positive affective experiences, meaning, and value congruence, alongside less frustration and constraint, which conceptually aligns with the affective or hedonic emphasis of AJS. Notably, the direct association between DBNSL and AJS remained significant after including W-BNS and WM, indicating that W-BNS should be interpreted as one proximal explanatory pathway rather than a full account of the DBNSL-AJS association. Given the cross-sectional, self-report design, these interpretations are limited to theory-consistent associations and statistical indirect effect estimates rather than evidence of causal mechanisms or temporal ordering.

### 5.3 Work motivation as a motivational pathway linking leadership and affective satisfaction

After controlling for gender, age, and academic rank, WM showed an independent and statistically significant indirect pathway in the association between DBNSL and AJS. The corresponding path pattern indicated that DBNSL was positively associated with WM and that WM was positively associated with AJS, suggesting that motivational functioning provided a partial statistical account of the DBNSL-AJS association and could be interpreted as a motivational pathway linking departmental need support with affective job satisfaction. The co-occurrence between managerial need support and stronger motivational functioning has been documented across contexts. In a self-determination framework that also considers pay and fairness, managerial need support has been positioned as a key condition associated with intrinsic motivation (Olafsen et al., 2015). Public sector samples likewise show positive associations among organizational and supervisor support, motivation, and engagement (Gillet et al., 2013). Transformational leadership research has further suggested that leadership-work attitude associations can be statistically decomposed at the level of basic need satisfaction (Kovjanic et al., 2012), and meta-analytic evidence indicates that more autonomously internalized motivation shows the strongest associations with satisfaction (Van den Broeck et al., 2021). Taken together, these findings align with a broader account in which supportive contexts correspond to motivational quality and favorable attitudes. Because WM in the present study was treated as a broader indicator of motivational functioning, evidence on intrinsic or autonomous motivation is interpreted as directional support rather than exact construct equivalence. As a supplementary leadership-process perspective, the goal clarification, barrier removal, and resource support embedded in DBNSL are directionally consistent with the observed positive association with WM. As a complementary work-design perspective, the motivational process of the job demands-resources model also helps interpret the association between WM and AJS by treating motivation as a key link between job resources and positive job attitudes. Together with SDT-based organizational research linking organizational factors, need-related processes, and work-related functioning (Gillet et al., 2012; Van den Broeck et al., 2010), this evidence supports interpreting WM as part of a broader motivational process rather than as an isolated correlate of AJS. Given the cross-sectional, self-report design, these interpretations are limited to theory-consistent associations and statistical indirect effect estimates rather than evidence of causal mechanisms or temporal ordering.

### 5.4 Integrating need satisfaction and work motivation in the serial pathway

After controlling for gender, age, and academic rank, the serial pathway DBNSL → W-BNS → WM → AJS was statistically significant, indicating that need satisfaction and work motivation jointly provided a theory-consistent statistical account of part of the association between department-based need-supportive leadership and affective job satisfaction. This serial pattern is consistent with self-determination theory, particularly basic psychological needs theory and organismic integration theory. Basic psychological needs theory supports the DBNSL → W-BNS segment by linking need-supportive contexts with satisfaction of autonomy, competence, and relatedness needs, whereas organismic integration theory supports the W-BNS → WM segment by explaining how need satisfaction is linked to internalization and higher-quality motivational functioning (Deci et al., 2001; Gagné and Deci, 2005). Teacher-focused research has also examined leadership and need satisfaction within a common framework and emphasized need satisfaction as a key correlational link in leadership-related differences in positive work functioning (Messmann et al., 2022). Regarding the need satisfaction-to-motivation segment, reviews of basic psychological needs theory and empirical studies across organizational settings provide convergent evidence that satisfaction of autonomy, competence, and relatedness needs is associated with more adaptive motivational functioning, including intrinsic or autonomous motivation (Vansteenkiste et al., 2020; Demircioglu and Chen, 2019). Related work has also examined stable sequences such as work characteristics → need satisfaction → autonomous motivation → employee functioning (Trépanier et al., 2015). Serial channels through which leadership relates to motivational quality have also been identified in more complex contexts; for instance, servant leadership, error management culture, and basic need satisfaction have been examined jointly in relation to motivational quality differences (Hudecek et al., 2024). Experimental studies have likewise tested sequences such as leadership → need satisfaction → motivational states → outcomes and reported differences following contextual manipulations (Kovjanic et al., 2013), which is directionally consistent with the serial association pattern observed here.

As a complementary work-design perspective, the job demands-resources motivational process also helps interpret the later segment of the pathway by treating motivational functioning as a key link between job resources and positive job attitudes (Bakker et al., 2014). Evidence on motivational profiles, SDT-based leadership samples, and work-focused SDT reviews further supports the view that need satisfaction, motivational quality, job satisfaction, and other favorable work states form a proximal system relevant to workplace functioning (Howard et al., 2016; Roche and Haar, 2020; Manganelli et al., 2018). Given the cross-sectional, self-report survey design, interpretations of the serial pathway are limited to theory-consistent associations and statistical indirect effect estimates rather than evidence of causal mechanisms or temporal ordering. Nonetheless, the DBNSL → W-BNS → WM → AJS decomposition provides a more systematized statistical account of the association between departmental need support and teachers’ affective job satisfaction.

### 5.5 Theoretical implications

This study strengthens the conceptual positioning of department-based need-supportive leadership in higher education. By locating need-supportive leadership at the departmental level, the study clarifies that leadership support in university settings can be understood not merely as a broad leadership style or a general perception of supervisor support, but as a theoretically specified form of social-contextual support grounded in self-determination theory. This positioning helps sharpen the conceptual boundary of DBNSL and highlights the department as a meaningful organizational level for studying university teachers’ work experiences. It also extends leadership-satisfaction research by shifting the theoretical focus from a general direct association between leadership and job satisfaction to a more differentiated explanation involving need-related and motivational processes.

The findings further suggest that the association between department-based need-supportive leadership and affective job satisfaction can be understood through distinguishable psychological and motivational layers. Work-related basic psychological need satisfaction represents a proximal psychological layer, whereas work motivation represents a motivational layer through which leadership-related experiences may be connected with teachers’ affective evaluations of work. In this sense, the study contributes to self-determination theory-informed organizational psychology by applying the sequence of need support, need satisfaction, motivational functioning, and affective work attitudes to Chinese university teachers in departmental governance contexts. Because the evidence is based on cross-sectional self-report data, these theoretical implications should be understood as implications derived from theory-consistent associations and statistical indirect effect estimates rather than evidence of causal mechanisms or temporal ordering.

## 6 Practical implications

These findings offer practice-oriented implications for departmental governance and faculty development in higher education. The practical focus is on translating department-based need-supportive leadership from an abstract principle into observable, trainable, and evaluable leadership behaviors. From an applied self-determination theory perspective, department leaders may support teachers’ affective work experiences by fostering autonomy, competence or structure, and relatedness in everyday governance, while also treating teachers’ need satisfaction and motivational functioning as process indicators for improvement.

For day-to-day implementation, departments can organize management practices around the three need-supportive dimensions of DBNSL. Autonomy support can be strengthened by establishing channels for participation and choice in task allocation and key decisions, clarifying available options and decision boundaries, and improving procedural transparency and predictability. Competence or structure support can be strengthened by providing specific and respectful development-oriented feedback and by ensuring that training, funding, and time resources are accessible for teaching and research tasks. Relatedness support can be strengthened through regular one-to-one meetings, mentoring arrangements, peer-collaboration mechanisms, and timely responses to teachers’ concerns. In addition, goal clarification, barrier removal, resource coordination, and supportive communication can help provide clearer organizational cues for teachers’ motivational functioning and affective job satisfaction.

To enhance evaluability, departments may develop management indicators that map onto the behavioral dimensions of DBNSL and incorporate them into department-chair training, feedback, and performance improvement routines. These indicators may inform the design of leadership-development programs or departmental intervention programs focused on autonomy support, competence or structure support, and relatedness support (Tafvelin et al., 2019). Repeated measurement of W-BNS, WM, and AJS each semester or academic year, combined with multi-source information such as supervisor ratings, peer feedback, and institutional process data, can be used to monitor patterns of change and identify priorities for improvement. Given the cross-sectional, self-report survey design, these implications should be interpreted as practice-oriented suggestions based on theory-consistent associations and statistical indirect effect estimates rather than evidence of causal mechanisms or temporal ordering.

## 7 Limitations and future directions

This study examined the association structure among department-based need-supportive leadership (DBNSL), work-related basic psychological need satisfaction (W-BNS), work motivation (WM), and affective job satisfaction (AJS) in a sample of Chinese university teachers. After controlling for gender, age, and academic rank, we estimated indirect effect patterns, including a serial indirect pathway, to describe an association structure consistent with the theorized ordering. Because the study relied on a cross-sectional, self-report survey design and because measurement and modeling choices entail methodological constraints, the findings should be interpreted as theory-consistent associations and statistical indirect effect estimates rather than evidence of temporal ordering or causal mechanisms. The main limitations and directions for future research are outlined below.

### 7.1 Limitations of measurement methods

All variables were measured using same-source self-report questionnaires, which may introduce common method variance and socially desirable responding. Although Harman’s single-factor test provided a coarse diagnostic, method variance and response-style effects cannot be fully ruled out. The Multidimensional Work Motivation Scale was adapted from a 7-point to a 5-point response format, and the resulting scores should not be compared directly with findings obtained using the original 7-point format. In addition, we used composite scores in regression models and PROCESS-based indirect effect estimation, rather than simultaneously modeling measurement error and structural paths at the latent-variable level. Moreover, measurement invariance was not tested; therefore, interpretations of mean differences across gender, age, and academic rank should be made cautiously. Future research should (1) incorporate teacher-leader paired data and multi-source indicators (e.g., peer or supervisor ratings and institutional process data), (2) use multi-wave or experience sampling designs to reduce same-source bias, and (3) test and report measurement invariance and cross-group comparability within a latent-variable framework.

### 7.2 Limitations in study design and causal inference

This study used a one-time cross-sectional survey design, collecting data on department-based need-supportive leadership, work-related basic psychological need satisfaction, work motivation, and affective job satisfaction at the same time point. This design allowed an initial test of a theory-consistent association structure, but interpretations remain limited to associations and statistical indirect effect estimates and do not constitute evidence of temporal ordering or causal mechanisms. The serial indirect pathway estimates also do not rule out reverse associations or alternative ordering models, and competing models were not systematically compared. Future studies should consider multi-wave longitudinal designs with cross-lagged tests, intervention studies, or quasi-experimental designs embedded in departmental management practice to examine whether changes in DBNSL, W-BNS, WM, and AJS show temporal alignment and to strengthen causal inference.

### 7.3 Limited adjustment for confounding factors

Although gender, age, and academic rank were controlled in the main analyses, unmeasured or insufficiently modeled confounders may still influence the estimated associations among department-based need-supportive leadership, work-related basic psychological need satisfaction, work motivation, and affective job satisfaction. For example, workload and role stress, organizational justice and procedural transparency, team climate and coworker support, stable individual characteristics (e.g., negative affectivity), and department-level resource differences may be related to both perceived leadership and satisfaction experiences. Future research should more systematically include key contextual and individual variables guided by the theoretical framework. Where the data structure permits, multilevel models or cluster-robust standard errors can be used to address the nonindependence of teachers nested within departments, thereby improving the robustness of standard error estimation and significance testing. In addition, robustness checks and sensitivity analyses may help evaluate how dependent the conclusions are on confounding assumptions.

### 7.4 Model robustness and hypothesis testing

Although confirmatory factor analysis supported good fit for the four-construct measurement model, hypothesis testing at the structural level relied primarily on composite-score regression and PROCESS-based indirect effect estimation. We did not conduct systematic comparisons among competing structural models or perform order-sensitivity tests, such as alternative mediator orderings, parallel versus serial specifications, or broader model sensitivity analyses. Therefore, the current evidence is best interpreted as associations and indirect effect estimates consistent with the theorized ordering rather than as proof of model uniqueness or optimality. Future studies could use structural equation modeling to estimate measurement and structural paths simultaneously, compare competing models in terms of fit and path stability, and strengthen robustness through alternative ordering tests, subsample replication, or cross-validation.

### 7.5 Sample and generalizability

The sample was drawn from in-service university teachers at a single institution, Hengyang Normal University, using non-probability convenience sampling, and participation was voluntary. Therefore, the findings should not be interpreted as nationally representative of all Chinese university teachers. The results are most applicable to university teacher populations in similar institutional and cultural settings, and generalization to other occupational groups, different types of educational institutions, or other countries and regions should be made cautiously. In addition, institutional type, regional context, resource structure, disciplinary composition, and departmental governance arrangements may shape leadership practices and teachers’ work experiences. Future research should replicate the model in multi-region and multi-institution samples, across different types of higher education institutions such as normal universities, comprehensive universities, research-intensive universities, and vocational colleges, and, where theoretically appropriate, in other occupational contexts to enhance the generalizability of the findings.

### 7.6 Future research directions and applications

Future work may advance this line of research in several integrated directions. Longitudinal tracking, cross-lagged designs, intervention studies, and quasi-experimental approaches could be used to examine the temporal alignment and stability of the theorized ordering among DBNSL, W-BNS, WM, and AJS and to compare alternative ordering models. Teacher-leader paired data, peer or supervisor ratings, institutional process indicators, and experience sampling could help reduce same-source bias and capture more context-sensitive changes in need satisfaction and motivation. Multi-department, multi-university, and cross-regional studies could further identify department-level differences and boundary conditions such as workload, organizational justice, psychological safety, and resource availability. In applied research, need-supportive management could be translated into evaluable departmental governance programs, with W-BNS and WM used as process indicators and AJS used as an outcome indicator for intervention evaluation.

## 8 Conclusion

This study examined the association structure between department-based need-supportive leadership and affective job satisfaction among Chinese university teachers. The findings indicated that department-based need-supportive leadership was positively associated with affective job satisfaction. This association included a direct component and could also be statistically decomposed into indirect effect estimates through work-related basic psychological need satisfaction and work motivation, including a serial pathway consistent with a need satisfaction to motivational functioning sequence. Because the direct association remained significant after both mediators were included, work-related basic psychological need satisfaction and work motivation should be interpreted as providing a partial statistical account rather than a full explanation of the DBNSL-AJS association. Theoretically, the findings support an SDT-informed account in which department-based need-supportive leadership is linked to teachers’ affective job satisfaction through need-related and motivational pathways. Practically, the findings point to the value of translating need-supportive leadership into observable departmental governance practices that support teachers’ autonomy, competence or structure, relatedness, and motivational functioning.

These conclusions should be interpreted in light of the study design. Because the study relied on cross-sectional, self-report questionnaire data, the findings should be understood as theory-consistent associations and statistical indirect effect estimates rather than evidence of causal mechanisms or temporal ordering. Same-source measurement and the single-institution sampling context also limit the generalizability of the findings. Future research may further test the proposed association structure using multi-wave longitudinal designs, teacher-leader paired data, multi-source measurement, intervention or quasi-experimental approaches, and multi-institution samples across different types of higher education institutions.

## Data Availability

The original contributions presented in the study are included in the article/Supplementary material, further inquiries can be directed to the corresponding author/s.
